# Short-term influence of cataract surgery on circadian biological rhythm and related health outcomes (CLOCK-IOL trial): study protocol for a randomized controlled trial

**DOI:** 10.1186/1745-6215-15-514

**Published:** 2014-12-29

**Authors:** Keigo Saeki, Kenji Obayashi, Tomo Nishi, Kimie Miyata, Shinji Maruoka, Tetsuo Ueda, Masahiro Okamoto, Taiji Hasegawa, Toyoaki Matsuura, Nobuhiro Tone, Nahoko Ogata, Norio Kurumatani

**Affiliations:** Department of Community Health and Epidemiology, Nara Medical University School of Medicine, 840 Shijocho, Kashiharashi, Nara, 634-8521 Japan; Department of Ophthalmology, Nara Medical University School of Medicine, 840 Shijocho, Kashiharashi, Nara, 634-8521 Japan; Center for Academic Industrial and Governmental Relations, Nara Medical University School of Medicine, 840 Shijocho, Kashiharashi, Nara, 634-8521 Japan

**Keywords:** Cataract surgery, Circadian rhythm, Depression, Sleep disturbance, Diabetes mellitus

## Abstract

**Background:**

Light information is the most important cue of circadian rhythm which synchronizes biological rhythm with external environment. Circadian misalignment of biological rhythm and external environment is associated with increased risk of depression, insomnia, obesity, diabetes, cardiovascular disease, and cancer.

Increased light transmission by cataract surgery may improve circadian misalignment and related health outcomes. Although some observational studies have shown improvement of depression and insomnia after cataract surgery, randomized controlled trials are lacking. We will conduct a parallel-group, assessor-blinded, simple randomized controlled study comparing a cataract surgery group at three months after surgery with a control group to determine whether cataract surgery improves depressive symptoms, sleep quality, body mass regulation, and glucose and lipid metabolism.

**Methods/Design:**

We will recruit patients who are aged 60 years and over, scheduled to receive their first cataract surgery, and have grade 2 or higher nuclear opacification as defined by the lens opacities classification system III. Exclusion criteria will be patients with major depression, severe corneal opacity, severe glaucoma, vitreous haemorrhage, proliferative diabetic retinopathy, macular oedema, age-related macular degeneration, and patients needing immediate or combined cataract surgery. After baseline participants will be randomized to two groups. Outcomes will be measured at three months after surgery among the intervention group, and three months after baseline among the control group. We will assess depressive symptoms as a primary outcome, using the short version geriatric depression scale (GDS-15). Secondary outcomes will be subjective and actigraph-measured sleep quality, sleepiness, glycated haemoglobin, fasting plasma glucose and triglyceride, low-density lipoprotein cholesterol, high-density lipoprotein cholesterol, body mass index, abdominal circumference, circadian rhythms of physical activity and wrist skin temperature, and urinary melatonin metabolite. Chronotype and visual function will be assessed using the ‘morningness-eveningness’ questionnaire, the Munich chronotype questionnaire, and the National Eye Institute Visual Function Questionnaire.

**Discussion:**

Although there are potential limitations due to the difference in duration from baseline survey to outcome measurements between two groups, any seasonal effect on the outcome measurement will be balanced as a result of continuous inclusion of participants through the year, and outcomes will be adjusted for day length at outcome measurements at analysis.

**Trial registration:**

UMIN000014559, UMIN Clinical Trials Registry, registered on 15 July 2014.

## Background

Light information is the dominant stimulus synchronising the master internal biological clock at the suprachiasmatic nuclei (SCN) with the external environment. The internal biological rhythm of humans is close to that of the earth’s rotation, according to a study of the rhythm of core body temperature independent of the external environment [1]. The misalignment of biological rhythm and external environment is associated with many health problems. Epidemiological studies among shift workers suggest that circadian misalignment is significantly associated with an increased risk of sleep disturbance [2], depression [3], obesity and metabolic syndrome [4, 5], diabetes [6, 7], ischaemic heart disease [8, 9], and stroke [10]. Light information modifies the timing of the internal biological rhythm according to the phase response curve to light [11]. Light exposure in the early morning (after core body temperature minimum) is responsible for the subsequent phase advance in melatonin secretion; in contrast, light in the evening (before core body temperature minimum) is responsible for the subsequent phase delay. Simultaneously, the amplitude of the internal biological rhythm is modified by light information [12].

In contrast to visual light information received by rod and cone cells of the retina and transmitted via the optic nerve, non-visual light information is mainly perceived by recently discovered intrinsically photosensitive retinal ganglion cells (ipRGCs), which contain melanopsin, and are transmitted to the SCN via the retinohypothalamic tract (RHT) [13]. The action spectrum for melatonin suppression among humans show a peak at a shorter wavelength (464 nm) than that for visual information (approximately 555 nm) [14].

Cataracts are a prevalent cause of visual impairment and a leading cause of blindness worldwide. The World Health Organisation reported that 33% of visual impairment (representing 95 million people) and 51% of blindness (representing 19.9 million people) are due to cataracts [15]. Among age-related cataract patients, the light transmission at the most sensitive spectrum for the photic entrainment of internal biological rhythms decreases from 82% at 10 years to 23% at 80 years [16]. Circadian misalignment because of the decreased input of light information caused by cataracts may explain the higher prevalence of depression among cataract patients [17], and the association of decreased light transmission by lens yellowing with sleep disturbance [18].

The research hypothesis of the present study is that cataract surgery, which removes the clouded lens and implants an artificial intraocular lens (IOL), will increase the input of non-visual light information and improve circadian alignment and its related health outcomes, such as depression, sleep disturbances, body mass regulation, and glucose and lipid metabolism [19].

The hypothesis is supported by studies of bright light intervention and light exposure in real-life situations. A Cochrane systematic review showed a significant reduction of depressive symptoms (standardised mean difference −0.20, 95% CI −0.38 to −0.01) from a meta-analysis of 18 randomized controlled trials (RCTs) among 505 patients with non-seasonal depression [20], and another systematic review also revealed the significant effectiveness of bright light intervention and light exposure among seasonal affective disorders (eight RCTs totalling 132 patients) [21]. In addition, recent RCTs have revealed the effectiveness of bright light therapy on depression, accompanied by improved sleep quality and internal biological rhythm. Bright light therapy (pale blue light at 7,500 lux for one hour) significantly decreased depressive symptoms compared with the placebo control group (red dim light at 50 lux) among 89 patients with non-seasonal depression, and was accompanied by an increase in salivary melatonin in the evening and better actigraph-measured sleep parameters [22, 23]. An intervention to increase light exposure by installing a ceiling light in the shared living room in group care facilities significantly decreased depressive symptoms, and increased total sleep time, as assessed by an actigraph, among elderly participants. In addition, a significant interaction of light intervention and melatonin administration on sleep efficiency was observed [24]. Higher daylight exposure increases melatonin secretion at night, according to an interventional study [25] and our population-based observational study [26].

Short-term and long-term influence of cataract surgery on depression and subjective sleep quality have been reported by some observational studies. Compared with depressive symptoms assessed before cataract surgery, significant decreases in depressive symptoms have reported at two months [27, 28], three months [29], and one year [30] after surgery. In addition, improved subjective sleep quality was reported at one month [31], two months [32, 33], and nine months [34] after surgery. However, evidence from RCTs on the effect of cataract surgery on depressive symptoms, sleep disturbance, and internal biological rhythms is lacking.

In contrast to bright light therapy conducted during the daytime, cataract surgery may increase the input of light information, not only in the daytime, but also during the night-time. Increased input of light information during the night-time may be deleterious to health outcomes. Indeed, we found significant cross-sectional association of increased light at night with increased prevalence of obesity, dyslipidaemia [35], sleep disturbance [36], and depression [37] in real-life situations among elderly individuals. These associations are supported by previous experimental evidence [38–40].

Here, we will conduct a parallel-group, assessor-blinded, simple RCT comparing the intervention group at three months after surgery with the control group at three months after baseline, to determine whether cataract surgery modifies the internal biological rhythm and improves its related health outcomes such as depression, sleep quality, body mass regulation, and glucose and lipid metabolism.

## Methods/Design

All intervention processes associated with the present study are conducted at Nara Medical University hospital. The protocol of this study has been registered at the University Hospital Medical Information Network Clinical Trials Registry (UMIN-CTR, identifier: UMIN000014559). The CLOCK-IOL (Cataract Surgery and Circadian Biological Rhythm among Japanese Older People with Cataract in Nara, Kansai Region: Influence of Intra Ocular Lens Implantation) study was approved by the institutional review board of Nara Medical University (approval number: 13-032). This research complies with the Declaration of Helsinki. Before enrolment to this study, we will obtain written informed consent from all participants.

**Figure 1:**
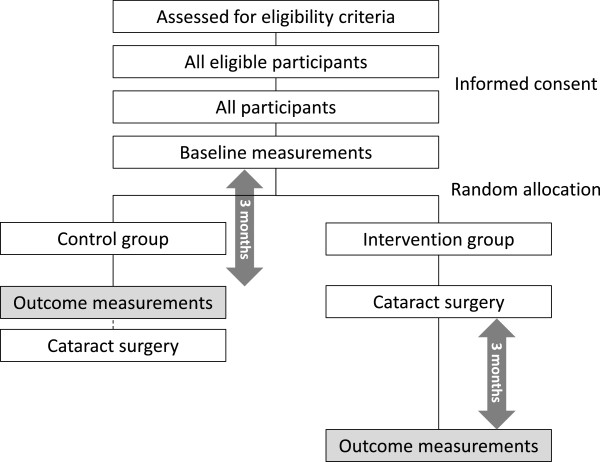
**Flow chart of participants.**

We will include participants continuously through the year with similar speed, and inclusion of the present study will finish at the same date as the started year. After baseline measurements, all participants will be randomly allocated to either the intervention group or the control group in a 1:1 ratio without restrictions such as blocking and stratification. The outcomes among the intervention group will be measured at three months after surgery. Among the control group, the outcomes will be measured at three months after baseline. The control group will receive delayed cataract surgery shortly after the outcome measurement (Figure 1).

### Participants

We will assess the eligibility of patients in Nara Medical University Hospital who have been diagnosed as having cataracts according to the following inclusion and exclusion criteria.

#### Inclusion criteria

The inclusion criteria for this study are as follows:

1. Patients scheduled for their first cataract surgery,
2. Aged 60 years or over, and
3. Diagnosed with cataracts of grade 2 nuclear opacification as defined by the lens opacities classification system III [41].

#### Exclusion criteria

The exclusion criteria for this study are as follows:

1. Major depression with current therapy,
2. Severe mental illness or dementia,
3. Severe corneal opacities with difficulty in assessment of lens opacity or fundal examination,
4. Glaucoma with a visual field deficit of at least mean deviation >14 dB (Humphrey perimeter),
5. Vitreous haemorrhage,
6. Proliferative diabetic retinopathy,
7. Macular edema,
8. Age-related macular degeneration,
9. Patients needing immediate cataract surgery, or
10. Patients needing combined cataract and glaucoma surgery or combined cataract surgery and vitrectomy.

### Intervention

Before the cataract surgery, the axial length of the eye will be measured with an A-scan UD-6000 (Tomey, Nagoya, Japan). Yellow aspherical IOL (SN60WF, Alcon, Fort Worth, Texas, United States), yellow spherical IOL (SN60AT, Alcon, Fort Worth, Texas, United States), or clear spherical IOL (SA60AT, Alcon, Fort Worth, Texas, United States) will be used for the cataract surgery. The appropriate power of the IOL will be estimated using the SRK/T formula for each IOL [42]. The kind of IOL used for the cataract surgery was randomly allocated to yellow spherical IOL, yellow aspherical IOL, or clear spherical IOL in a 1:1:2 ratio. After phacoemulsification with a small incision, IOL will be implanted.

### Patients of cataract surgery for both eyes

We include participants undergoing cataract surgery for single and both eyes. The surgery for both eyes among the intervention group will be completed within the same time-scale, one to two weeks, and the same kind of IOLs will be used for both eyes. Outcomes will be measured at three months after the latest surgery.

### Primary outcome

We will assess depressive symptoms as a primary outcome of the present study using the short version geriatric depression scale GDS-15. Prevalence of depression, median of GDS-15, and the mean value of difference between baseline and three months later will be compared between the intervention group and the control group.

### Secondary outcomes

As secondary outcomes, we will measure subjective and actigraph-measured sleep quality, sleepiness, glycated haemoglobin (HbA1c), fasting plasma glucose (FPG), triglyceride (TG), low-density lipoprotein cholesterol (LDL-C), high-density lipoprotein cholesterol (HDL-C), body mass index (BMI), abdominal circumference, circadian rhythm of physical activity and wrist skin temperature, urinary melatonin metabolite, chronotype, post-illumination pupil response (PIPR), visual acuity, and subjective visual function.

### Self-reported questionnaires

Depressive symptoms are measured using GDS-15; a self-administered questionnaire consisting of 15 items [43]. The sensitivity and specificity of GDS-15 compared with diagnosis according to the Diagnostic and Statistical Manual of Mental Disorders (fourth edition) was 92.7% and 54.8% with a cut-off point of four out of five, and 84.8% and 67.7% with a cut-off point of six out of seven, respectively [44]. According to a meta-analysis about the validity of GDS-15, the mean sensitivity was 0.805 and the mean specificity was 0.750, respectively [45]. A higher score on the GDS-15 was significantly associated with self-report and clinician-administered measures of suicidal ideation [46] and higher rates of suicide [47]. Subjective sleep quality and daytime sleepiness are assessed using the Pittsburgh Sleep Quality Index (PSQI) [48] and the Epworth Sleepiness Scale (ESS) [49, 50], respectively. Chronotype and subjective visual function are determined by the ‘morningness-eveningness’ questionnaire (MEQ) [51], the Munich Chronotype Questionnaire (MCTQ) [52], and the National Eye Institute Visual Function Questionnaire (NEI VFQ25) [53, 54].

### Analysis of venous blood sample

Overnight fasting venous blood samples will be analysed at a commercial laboratory (SRL Co. Inc., Tokyo, Japan) using standard clinical chemistry analysis to determine the concentrations of HbA1c, FPG, TG, LDL-C, and HDL-C.

### Morning spot urine

We will measure 6-sulfatoxymelatonin (aMT6-s) in a morning spot urine sample. Peak nocturnal plasma melatonin is significantly associated with aMT6-s in subsequent morning spot urine (r = 0.69) [55, 56]. Urinary aMT6-s concentration will be measured at a commercial laboratory (SRL Co. Inc. Tokyo, Japan) using an ELISA kit (RE54031; IBL International, Hamburg, Germany).

### Actigraph

Objective sleep will be measured by an actigraph (ActiSleep-BT Monitor; ActiGraph Inc., Florida, United States), worn on the non-dominant arm for five days, including weekdays and a weekend. Participants will be instructed to keep a standardised sleep diary logging bed time and rising time. Sleep parameters such as total sleep time, sleep efficiency, sleep onset latency, and wake after sleep onset will be calculated with ActiLife 6 (ActiGraph Inc. Florida, USA). A validation study of this device showed moderate to high agreement with sleep parameters as measured by polysomnography [57].

### Circadian rhythm of physical activity and wrist skin temperature

Invasively measured biomarkers usually used in laboratory settings, such as fluctuation of plasma cortisol, melatonin, and rectal temperature, are difficult to conduct because they disturb the normal life of participants. To assess the influence of cataract surgery on internal biological rhythms using non-invasive methods, we will measure the phase and amplitude of circadian activity rhythm and the wrist skin temperature.

According to large-scale prospective cohort studies, decreased amplitude, later phase, and decreased robustness of circadian activity rhythm showed a significantly higher hazard ratio for incidents of cognitive disorders, cancer mortality, and all-cause mortality [58–60]. Wrist temperature reveals heat loss from arteriovenous anastomoses at the skin [61], and shows a mirror image of core body temperature [62] and blood pressure [63, 64]. The validity of using wrist skin temperature as an acceptable measure to assess circadian phase is indicated by the significant correlation of dim light melatonin onset and the increase of wrist skin temperature in the evening in real-life situations (r = 0.76) [65]. The wrist skin temperature at inside of the wrist, near the radial artery of the dominant arm, will be measured using a temperature data logger (Thermochron iButton; Maxim/Dallas, Dallas, Texas, United States) at three-minute intervals [62].

### Post-illumination pupil response

By pharmacological blockage of the rod and cone cells, the melanopsin-associated ganglion cell response is isolated as a slow, maintained depolarisation to light stimulation, which repolarises slowly after light-offset *in vitro* [66]. This PIPR is indicated as an index of the sensitivity of the melanopsin-containing ipRGC pathway [67]. In the present study, baseline and sustained pupil diameter after blue and red light stimulation will be measured. Baseline pupil diameter is the average pupil diameter in a seven-second period before light onset. Sustained pupil diameter is the average from 10 to 40 seconds after light offset. PIPR (mm), PIPR change (%), net PIPR (mm), and net PIPR change (%) will be calculated as follows [68]:


### Other variables

Using a standardised questionnaire, trained researchers will interview participants about basic characteristics, such as age, gender, smoking and drinking habits, past history of cardiovascular events, stroke, cancer, medication, household income, years of education, and history of shift work. BMI will be calculated as weight per height (kg/m^2^). Waist circumference will be measured at the level of the umbilicus, in the standing position.

### Sample size

According to the HEIJO-KYO study [26], a community-based cohort study among the elderly (mean age ± standard deviation: 72 ± 7.1) in the same district as the present study, the prevalence of depression was 20.0% (101 out of 506) [37]. To detect its 7% difference as significant at a two-sided α level of 5% with a power of 80%, it was calculated that 475 participants in each group would be required. Assuming a dropout of 5%, we estimated that a total of 1,000 participants would be needed.

### Randomization and masking

Allocation concealment will be maintained by central randomization using computer-generated random sequences, independent of care-providers. The results of the allocation will be masked from the assessors of the outcomes, but will be available to both the care providers and the participants.

### Statistical analysis

We will compare the outcomes between the control group and the intervention group based on the intention-to-treat principle. For missing values due to loss to follow-up after baseline measurement, we will impute baseline data using the last observation carried forward (LOCF) method. For continuous variables with normal distributions, the mean and standard deviation will be reported. For variables not distributed normally, the median and interquartile range will be reported. Means, medians, and proportions will be compared using the t test, the Mann-Whitney U test, and the χ^2^ test, respectively. We will use analysis of covariance to estimate adjusted mean values and 95% CIs. The prevalence of the two groups will be tested using multivariate logistic regression analysis. To assess the phase, amplitude, and robustness of the circadian rhythm, we will use the cosinor model [69], the sigmoidally transformed cosine model [70], and the generalised additive model [71]. We will conduct a subgroup analysis at baseline according to the severity of cataract and chronotype.

## Discussion

To conduct an RCT assessing the long-term effect of cataract surgery is ethically difficult, because the effectiveness of cataract surgery for visual acuity is established. To assess the effect of cataract surgery after three months with minimum disadvantage to the control group due to delayed surgery, we have potential limitations due to the difference of duration from baseline survey to outcome measurements between the two groups. Compared with the control group, the intervention group will spend a longer time from baseline measurement to outcome measurement due to the duration between baseline and surgery, and it may distort the results because of a seasonal effect on outcomes. However, we will continuously include participants with a similar speed though the year, and will finish this study at the same date as started in the previous year. As a result, the number of overestimated outcomes and underestimated outcomes due to the seasonal influence will be balanced between the two groups. In the statistical analysis, we will compare the outcomes adjusted for seasonal variables, such as day length at outcome measurement, using ANOVA for continuous variables and multivariate logistic regression analysis for categorical outcomes.

## Trial status

At the time of submission, the study protocol has been fixed and registered for clinical trial registration, but the recruitment of participants has not started. Recruitment of participants is expected to begin August 2014.

## Abbreviations

aMT6-s: 6-sulfatoxymelatonin
BMI: Body mass index
FPG: Fasting plasma glucose
GDS-15: The short version geriatric depression scale
HDL-C: High-density lipoprotein cholesterol
IOL: Intraocular lens
ipRGCs: Intrinsically photosensitive retinal ganglion cells
LDL-C: Low-density lipoprotein cholesterol
PIPR: post-illumination pupil response
RCTs: Randomized controlled trials
SCN: Suprachiasmatic nuclei
TG: Triglyceride.
